# Supplementary material for: Are carbon emissions trading and green financial instruments synergistic? -Comprehensive quantitative research based on content analysis
Source: PLoS One. 2024 Mar 7;19(3):e0298601. doi: 10.1371/journal.pone.0298601 (PMC10919601; doi:10.1371/journal.pone.0298601)
Supplement: S1 Data — (ZIP) [file pone.0298601.s001.zip › Data/地级市Z_F工作报告.zip!/╕ú╜¿/╧├├┼╩╨/└┤╫╘╣1⁄2╓┌║┼í╛┬φ┐╦╩2╛▌═°í┐_2014.docx]

各位代表:

　　现在，我代表市人民政府向大会报告工作，请予审议，并请各位政协委员和其他列席人员提出意见。

　　一、去年工作回顾

　　2013年，在市委的正确领导下，我们认真贯彻落实党的十八大精神，大力推进综合配套改革，全力实施跨岛发展战略，科学编制美丽厦门战略规划，更加注重发挥优势、扬长避短，更加注重优化结构、提升质量，更加注重改善民生、维护稳定，更加注重转变观念、改进作风，较好完成全年各项任务。实现地区生产总值3018亿元，增长9.4%；财政总收入和地方级财政收入分别为825亿元和491亿元，增长11.6%和16%,财政总收入占生产总值比重上升至26.9%;城镇居民人均可支配收入和农民人均纯收入分别为41360元和15008元，增长10.1%和11.5%；居民消费价格指数低于全国全省平均水平；万元生产总值耗电、耗水分别下降1.6%和1.2%。

　　一年来主要工作和成效是:

　　（一）扎实推进优化升级，经济发展方式加快转变

　　现代服务业加快发展。第三产业增加值1558亿元，增长7.7%，占地区生产总值51.6%，三产税收比重55.5%。社会消费品零售总额975亿元，增长10.5%。举办展览184场、会议4146场，投洽会、医博会等成果丰硕，石材展、佛事用品展规模全球最大，成为中国会展名城。旅游人气居全国前列，“吃住行游购娱”六要素整体提升，接待国内外游客4664万人次，总收入621亿元，分别增长13.1%和15%。空港旅客吞吐量1975万人次，增长13.8%。港口集装箱吞吐量801万标箱，增长11.2%，增幅居全国沿海主要港口第二。全市金融机构本外币存、贷款余额分别为6381亿元和5844亿元，增长16.6%和14.4%。6家小额贷款公司开业，两岸股权交易中心成立。跨境人民币结算金额累计突破1300亿元，成为两岸人民币现钞调运主渠道。厦门石油交易中心交易额突破900亿元。国内首个游艇水上保税仓正式运营。文化产业增加值超过260亿元，增长20%。

　　先进制造业实力增强。规模以上工业企业达1664家，总产值4678亿元，增长13.1%，其中产值超百亿元企业8家、超十亿元企业63家。电子、机械两大支柱产业分别实现产值1837亿元和1196亿元，厦门成为触控屏组件全球最大研发生产基地。生物医药产值超过200亿元，增长30%。支持企业完成技改投资201.8亿元，增长15%。获评首批全国质量强市示范城市，主导或参与制定国家标准、行业标准57项，质量竞争力指数保持全国领先。

　　创新驱动能力不断提高。新认定高新技术企业104家，总数达820家。规上高新技术企业实现产值2099亿元，占规上工业总产值44.9%。设立先进复合材料产业技术研究院等科研机构，获批建设分子疫苗学和分子诊断学等10个国家和省重点实验室，135个项目获国家科技计划立项。建立卫星导航等12个产业创新联盟，路达工业被认定为国家级企业技术中心，金牌橱柜入选国家级工业设计中心。国家知识产权局在厦设立专利信息服务中心，全市发明专利授权8255件，每万人拥有发明专利9.17件，获评国家知识产权示范城市。1700多名海内外高层次人才申报“海纳百川”计划，已入选200名。

　　智慧名城创建取得实效。加快“三网融合”，推进政务、医疗、交通、教育等云计算应用，成为国家下一代互联网示范城市。与工信部、省政府共建中国软件名城。软件园二期入驻企业563家，实现收入318亿元，成为国家新型工业化产业示范基地。软件园三期核准入驻企业243家，起步区30万平方米研发楼建成投用。软件和信息服务业实现收入591.7亿元，增长28.3%。

　　（二）扎实推进跨岛发展，城乡一体统筹力度加大

　　基础设施建设克难而进。厦深铁路建成通车，厦门每天通行动车98对、普速列车14对，运送旅客超过10万人，东南沿海重要铁路枢纽地位更加凸显。从思明老城区到火车北站、全程31公里24个站点的地铁1号线正式开工。翔安机场选址得到空军总部认可，前期工作取得实质性进展。高崎机场4号航站楼基本竣工，海沧隧道连接线工程进展顺利。总投资57亿元、全长9.3公里的厦漳跨海大桥建成通车，本岛到漳州开发区车程缩短1小时。库容1.2亿立方米、向我市日供水50万立方米的长泰枋洋水利枢纽工程加快推进，工可和库区移民安置规划获批。扩建海沧、翔安、高殿水厂和集美污水处理厂。改造疏浚排洪沟、积水点60处，防洪排涝能力增强。建成6座加气站，促进汽车燃油改燃气。新建6个输变电工程，第4条220千伏线路进岛。全年完成重点项目投资863亿元，占全市固投近65%，超过年度目标14个百分点。

　　城市功能品位不断提升。岛外新城完成投资507亿元，增长19.7%。杏林湾商务营运中心投用，灵玲国际马戏城主体建成，方特梦幻王国开业。灌口、东孚、汀溪、新圩4个小城镇建设加快。拆除非法占地违法建设39.3万平方米，岛内主城区道路清洗覆盖面扩大。筼筜湖综合整治进展顺利，鼓浪屿申遗促进环境综合整治和风貌建筑保护利用。思明、海沧“美丽厦门共同缔造”试点取得实效。

　　“三农”工作体现更多实惠。建成现代设施农业近6500亩，新建“菜篮子”直控基地16个，累计达79个，猪肉、蔬菜自给率分别为40%和45%。银鹭、银祥、中盛等33家农业产业化龙头企业带动近8万农户，实现产值342亿元，增长12.9%。实施新村建设、老区山区村改造项目32个，建成村道48 公里、管沟36公里，配套路灯2000多盏、篮球场32个、户外健身设施61套，村容村貌明显改观。行政村通自来水率和农家书屋设置率均为100%，有线电视入户率99.6%。转移农村富余劳动力近2万人，农民财产性收入继续增加，人均纯收入保持全省第一。

　　（三）扎实推进改革开放，发展动力活力不断增强

　　综合配套改革持续深化。国家有关部委在厦开展20多项改革试点，综合配套改革试验土地专项方案获批，16项省级行政审批职权下放我市。启动商事登记制度改革，170项前置审批事项改为证照分离、宽进严管。实施营改增试点，减轻企业税负16亿元。改革医药卫生体制，全面取消公立医院药品加价。加强政府性债务管理，债务率控制在国家规定的安全范围之内。

　　外经外贸工作富有成效。实现进出口总额841亿美元，增长12.9%，其中出口524亿美元，增长15.3%，外贸综合竞争力稳居全国百强城市前5位。推进无纸化通关，提高了办事效率，降低了企业报批成本。建成5个外贸公共服务平台，出口基地范围从15类增至21类。设立境外网点65个，境外投资额6.4亿美元，带动出口超过20亿美元。实际利用外资18.7亿美元，增长5.5%，新批外商投资项目331个。

　　对外影响力进一步扩大。入选对外开放金牌城市，成为外籍人才眼中最具吸引力城市。成功举办国际海洋周、国际友城市长论坛、34国驻华外交官“厦门行”。与塔吉克斯坦首都杜尚别缔结友城。新增国际航线2条，总数达18条。授予16位外国专家白鹭友谊奖，爱乐乐团圆满完成“土楼南洋行”。同时，厦漳泉同城化稳步推进，援藏援疆援甘援宁等对口帮扶取得实效。

　　（四）扎实推进交流合作，对台前沿优势日益凸显

　　经贸合作更加紧密。实现对台进出口总额81亿美元，增长 5.8%。实际利用台资3.9亿美元，增长29.9%，新批台资项目138个（含第三地）。新增赴台投资项目8个，三安光电并购璨圆光电，成为大陆赴台投资最大项目。闽台云计算产业示范区揭牌。海西首家两岸合资证券投资基金管理公司成立，厦台跨境人民币结算代理清算协议签约银行增至28对56家。两岸贸易中心挂牌，67家企业和商协会入驻。台湾水果、大米等货物进口量稳居大陆首位。

　　直接往来更为便捷。开展两岸交通物流信息平台建设试点，设置两岸通信业务出入口，启用五通码头新通关大厅。来厦省外人员赴金门旅游由1日延至2日。厦门至台北、台中、高雄保持每周35个空中航班。厦金航线保持每日36个航班，全年运送旅客125.6万人次。

　　交流交往更趋活跃。第五届海峡论坛发布促进两岸交流合作政策措施31项，累计近200项，每届参会台胞均超万人，拓宽了交流领域，增强了合作实效。举办台交会、文博会、图交会等重大活动30多场，影响力进一步提升。“乡音之旅”地方戏剧交流团赴台巡演，增进基层民众的文化认同。新设15个两岸交流合作基地或办事机构，为扩大交流创造了条件。

　　（五）扎实推进民生改善，和谐社会构建成效明显

　　社会事业发展全面提速。新建公办幼儿园18所、中小学项目34个，分别增加学位5490个和11000个，进城务工人员随迁子女接受公办义务教育比例提高至82.5%。面向全国招聘中小学教师1464名，成立中高职集团化办学教育联盟，理工学院成为省重点建设高校。隆重纪念集美学校建校100周年，大力弘扬嘉庚精神。儿童医院、海沧医院二期、第二医院三期等项目进展顺利。继续稳定低生育水平。成功举办全国中小学生艺术展演、中国国际钢琴比赛，大型舞蹈《沉沉的厝里情》等获国家级奖项，引进中演、保利集团分别经营闽南大戏院和嘉庚剧院，建成自助图书馆55个，获评国家公共文化服务体系示范区、文化和科技融合示范基地。厦门卫视在全国9省市落地。表彰一批优秀社科成果。举办第19届市运会，在第12届全运会上获得7枚金牌，厦门国际马拉松连续7年获评国际田联路跑金牌赛事。全市各区连续3届获评省级双拥模范城。民族宗教、人防、海防、侨务、科普、气象、防震、档案、地方志、妇女儿童、青年、老龄、残疾人、慈善等工作，都取得新成效。

　　民生保障水平不断提高。为企业招聘应届毕业生提供社保补贴，为就业困难人员开发公益性岗位，全年新增就业18万人。企业退休人员基本养老金上调310元，增量居全国首位。城乡居民医保财政补助标准提高至390元，高于国家标准。职工医保、城乡居民医保最高支付限额分别为50万元和45万元。143家村卫生所开通医保刷卡。厦门社保卡实现全省同城结算。岛外各区建成综合社会福利中心。新建5家养老机构，增加床位1510张。基本建成保障性住房1.4万套，动建1.1万套。完成首批特困家庭危房改造。更新和增投公交车240辆，新开或优化公交线路59条。水果和畜产品经农业部例行监测，合格率名列全国前茅。

　　社会治理能力持续提升。连续3届获评全国社会治安综合治理优秀城市，捧得“长安杯”。健全治安防控体系，刑事案件下降16%。大力排查化解信访积案，完善矛盾纠纷多元调解机制，建成行业性、专业性调委会282个。妥善处理快速公交放火案，及时救治伤员、安抚家属、侦破案件。建成网格化信息平台223个，城市社区实现全覆盖，成为全国社区治理和服务创新实验区。开展安全生产大检查和重点整治百日行动，总体状况持续好转。注册志愿者44万人，城市文明程度指数保持全国前列。

　　生态文明建设保持领先。完成4个国家级生态区创建，实现国家级生态镇全覆盖。治理水土流失1.7万亩，造林绿化2.1万亩，建成一批公园绿地，空气质量优良率93.4%，获评国家森林城市。海域清淤1850万立方米，成为首批国家级海洋生态文明建设示范区。在饮用水源地设立水质检测站点。汀溪水库水源保护区生猪退养全部完成。对全市雨污合流排放口实施截流集中处理。蝉联国家节水型城市。西部垃圾焚烧发电厂试运行，东部固废中心周边环境逐步改善。加强机动车尾气治理，成为全国首批十大低碳交通试点城市。

　　（六）扎实推进自身建设，行政效能水平有效提升

　　深入开展党的群众路线教育实践活动，坚持“四下基层”，严格落实中央八项规定和国务院“约法三章”，市级会议、文件大幅减少，“三公”经费支出减少13.5%，清退办公用房2.6万平方米，机构编制得到有效控制。办理人大议案5件、建议272件，办理政协提案589件，满意率分别为98.2%和94.3%。提请人大审议法规草案6件，制定和修订规章3件，废止和宣布失效市政府规范性文件147件。精简市级行政审批事项96个，减少21.9%。647个审批服务事项进驻行政服务中心，71%办件实现即来即办，效能建设不断增强，连续3年绩效考评全省第一。强化监察、审计工作，加强廉政、法治建设，获评全国法治城市创建先进单位。

　　各位代表！面对复杂多变的外部环境和转型升级的内在压力，我们千方百计稳增长，坚定不移调结构，做到稳中有进、稳中有为，成绩来之不易。这是在市委坚强领导下，在人大代表、政协委员有效监督和大力支持下，全市人民齐心协力、艰苦奋斗的结果。在此，我谨代表市人民政府，向全市人民，向全体人大代表和政协委员、各民主党派、工商联、各人民团体、无党派人士、离退休老同志和社会各界人士，向中央和省驻厦单位、驻厦部队、武警官兵和公安干警，向来厦的投资者和劳动者，向关心支持厦门发展的港澳同胞、台湾同胞、海外侨胞和国际友人，表示衷心的感谢和崇高的敬意！

　　同时，我们也清醒认识到，当前发展还存在不少困难和问题，主要是:地区生产总值、固定资产投资增速减缓，经济增长动力减弱；资源与环境硬约束日益趋紧，劳动力成本上升，转型发展压力增大；经济特区政策优势弱化，产业整体竞争力不强，特色化、差异化竞争优势尚未形成；公共服务和民生保障与人民群众的期待仍有差距，城市综合管理能力还需加强，停车难、交通拥堵现象亟需缓解；审批环节多、效率低的问题亟待改进，干部队伍“四风”方面问题还不同程度存在，工作水平和能力有待提高。我们要正视这些问题，认真加以解决。

　　二、努力为建设美丽厦门开好局起好步

　　2014年，是完成“十二五”规划的关键一年。市委十一届六次全会讨论通过的美丽厦门战略规划，把中央大政方针落实到厦门实践，把厦门建设发展融入到国家战略，明确提出两个百年愿景、五个城市目标、三大发展战略、十大行动计划和共同缔造方案。建设美丽厦门，是我市实现科学发展的战略抉择，是转型所需、使命所系、民心所盼。我们要强化规划引领、落实五位一体、坚持循序渐进、推进共同缔造，坚定不移地向着美丽中国的典范城市和展现中国梦的样板城市迈进！

　　政府工作总体要求是:全面贯彻党的十八大、十八届二中、三中全会和习近平总书记系列讲话精神，坚持稳中求进、改革创新，以全面深化改革为动力，以建设美丽厦门为抓手，统筹推进稳增长、促转型、惠民生、控风险，着力提高发展的质量和效益，促进经济持续健康发展、社会和谐稳定。

　　经济社会发展主要预期目标为:地区生产总值增长10%左右，规模以上工业增加值增长11.5%，全社会固定资产投资增长15%，外贸出口总额增长11%，实际利用外资与上年持平，财政总收入和地方级财政收入分别增长10%和11%，城镇居民人均可支配收入和农民人均纯收入分别增长10%和11%，居民消费价格涨幅控制在3.5%以内，完成国家和省下达的节能减排任务。

　　实现上述目标，必须牢牢把握四项原则:一是坚持稳中求进，始终把质量和效益作为经济社会发展的基本取向，理性对待高速增长转向中高速增长的新常态，既以质量提升城市核心竞争力，又保持科学合理的增长速度，加快形成质量型增长、内涵式发展的良好态势；二是坚持改革创新，始终把先行先试作为经济特区的核心使命，以改革增创优势，以创新引领发展，加快建立系统完备、科学规范、运行有效的制度体系，为科学发展提供强大的不竭动力；三是坚持民生优先，始终把为人民谋利益作为政府工作的出发点和落脚点，突出保障基本民生、底线民生、热点民生，切实提升基本公共服务均等化水平，让全体市民过上更加美好的幸福生活；四是坚持保护环境，始终把生态文明建设融入经济社会发展的各领域，进一步形成尊重自然、顺应自然、保护自然的良好氛围，建设地绿水净、天朗气清、惠风和畅的美丽家园。

　　重点做好八个方面工作:

　　（一）着力优化产业结构，进一步打造特区经济升级版

　　实现有质量、有效益、可持续的发展，根本途径是加快转变发展方式，关键是深化产业结构战略性调整。

　　大力发展现代服务业。启动会展中心四期建设，扩大展馆面积，为更多国内外大型展会提供平台，运用市场手段提升展会规模效益。围绕“大旅游”产业链，培育海峡旅游、滨海旅游和闽南文化旅游三大品牌，大力推进邮轮母港建设，争取列入邮轮旅游发展实验区，推动旅游业成为重要支柱产业。促进文化与会展、旅游、科技融合发展，重点扶持创意设计、影视动漫、数字内容等文化产业。加快建设东南国际航运中心，着重发展口岸物流、第三方物流、城际配送物流，推动区域物流联动发展。做强两岸股权交易中心，扩大两岸跨境人民币结算代理行清算群规模。筹备设立大陆首家两岸合资全牌照证券公司。支持金融产品创新，促进金融服务实体经济发展。做大厦门石油交易中心，培育输台中药材等大宗商品交易市场。鼓励承接离岸服务外包。加快打造总部经济集聚区，吸引更多总部企业落户。

　　加快发展先进制造业。继续支持工业技术改造，抓好一批重点技改项目，提高企业生产自动化水平。加强要素保障，鼓励有市场、有潜力的重点企业增资扩产。大力引进一批带动性强、效益好的龙头项目，提升产业配套能力，促进主导产业高端化、传统产业新型化、新兴产业规模化。推动企业管理现代化，提升企业整体素质和市场竞争力。整合工业园区，规划建设集美机械工业集中区三期，加快科技创新园、火炬同安基地、海沧生物医药园区等配套建设，促进优势产业集聚发展。

　　积极创建国家信息消费示范城市。完善信息基础设施，推进宽带厦门工程，实现4G网络基本覆盖。加快软件园三期建设，重点发展云计算、北斗卫星应用、大数据、电子商务等产业，引进智能终端生产企业。落实智慧名城行动计划，创新“以用兴业”发展模式，实施数字家庭、智慧社区、智能交通等示范工程，推广物联网技术，形成研发、生产、应用、服务“四位一体”产业体系。

　　加快建设创新型城市。构建以企业为主体的技术创新体系，重点支持产业化基地、科技孵化器、重大科技创新平台建设，吸引央企、跨国公司的研发机构落户。培育软件与信息技术、生物医药、新材料、节能环保等重点产业集群。促进创新要素向产业集聚，推动科技成果产业化，以科技创新引领产业转型升级。深入实施“海纳百川”人才计划，培养和引进高端技术研发、产业领军人才，营造尊重知识、尊重创造的良好氛围。

　　做精做优都市现代农业。抓好农业科技园、农民创业园建设，大力发展高科技种苗业、设施农业，提升农产品精深加工水平，拓展休闲农业与旅游农业。推动农业规模化生产经营，提高土地生产效益。培育扶持农业产业化龙头企业，带动更多本市及周边地区农户。

　　（二）着力全面深化改革，进一步增创特区发展新优势

　　弘扬敢闯敢试、先行先试精神，坚持创新体制机制与争取政策突破并举，深化综合配套改革，为特区发展增添动力。

　　深化行政管理体制改革。完成新一轮政府机构改革，优化机构设置、职能配置、工作流程。大幅减少行政审批事项，压缩和规范自由裁量权，推行并联审批，缩短审批时限。落实好商事登记制度改革后续工作，建立监管联动机制和问责制，优化营商环境，激发市场活力。整合职能相近的事业单位，逐步取消学校、科研院所、医院的行政级别。

　　加快经济领域改革。发展混合所有制经济，完善公司制股份制改革，推动国有企业在改制上市、兼并重组、项目投资过程中引入民间资本。改革国有资本授权经营体制，以管资本为主加强国有资产监管。推进投融资体制改革，优化市对区财政体制，防控政府性债务风险。扩大营改增试点。完善促进信息消费、战略性新兴产业发展的体制机制。

　　拓展社会领域改革。建立城乡基础设施一体化的建设和管理体制，促进优质公共资源向岛外和农村延伸。建立城乡统一的建设用地市场，健全农民权益保障机制。完善科技、文化、教育、医药卫生体制机制，深化就业和社保制度改革。加强政务诚信、商务诚信、社会诚信建设，逐步完善诚信体系。

　　（三）着力扩大对外开放，进一步提高开放型经济水平

　　以全球视野、双向思维，不断加快城市国际化步伐，推动开放朝着优化结构、拓展深度、提高效益方向转变。

　　争取设立自由贸易园区。整合优化海沧保税港区、象屿保税区、保税物流园区等海关特殊监管区。探索实施“一线放开，二线安全高效管住”监管模式，建立统一高效的口岸联检机制。对外商投资实行准入前国民待遇加负面清单管理模式，扩大服务业开放领域，加快形成高标准、便利化的投资贸易规则体系。

　　增创开放发展新优势。统筹全市招商资源，建立专业化招商引资机制。瞄准国内外技术和管理水平先进的企业，加大推介力度，发挥引资综合优势，实现吸引资金、技术、智力有机结合。支持企业开拓国际市场，做精做优重点出口产品。推动酒类、水果、进口仓储等公共服务平台建设。鼓励企业开展跨国经营，建立营销服务网络。加强与国外友城合作交流，组织开展各类对外经济文化活动，提升城市国际知名度。

　　大力推进区域发展同城化。主动发挥推动、引领和服务作用，加快厦漳泉接壤区规划和项目对接，推进城际轨道交通等前期工作。探索异地共建产业园区新机制，加强产业对接，谋求差异发展。深化与对口支援地区经济技术合作。

　　（四）着力推进跨岛发展，进一步提升城市承载力宜居度

　　实施主体功能区战略，有效优化城市空间，加快解决城市化过程中面临的突出问题，提升城乡科学管理水平。

　　提升岛内拓展岛外。岛内按照降低开发强度、降低建设密度原则，实行最严格的建设规模控制，加快二产转移，推进功能疏解，提升环境品质。建好城市自行车道和慢行道，完善五缘湾、湖边水库等片区配套。岛外继续推进四大新城核心区起步区建设，加快聚集优质教育、卫生、文化等资源，力争公共服务设施建设取得新突破。

　　力推重大基础设施建设。地铁工程投资大、工期长、难度高，务必精心组织1号线的隧道开凿和站点设计，尽量减少施工对市民交通和生活影响。加快2号线、3号线和岛外环湾线前期工作，力争早日开工。统筹推进翔安机场立项报批、规划设计、填海造地。加快高崎机场4号航站楼装修工程。确保火车站改造如期竣工。全面建成厦成高速、海翔大道，加快建设海沧隧道、莲岳隧道、厦安高速互通、国道324改线工程。建成远海自动化码头，抓紧扩建厦门港主航道四期工程，启动海沧港南作业区前期工作，推动东渡港区1-4#泊位迁移。完成高集、马銮海堤开口改造。建成莲花水库。大力推进长泰枋洋水利枢纽工程移民安置点和引水隧洞建设，确保大坝按时动工。继续做好抽水蓄能电站前期工作，加快岛内高压线缆化进程。

　　增强防灾减灾能力。完善建成区防洪排涝功能，实施围里、岭兜等防涝改造。加强高层建筑、人员密集场所等重要区域火灾防控，建成新一代天气雷达，提高极端天气、地质灾害应对处置能力。细化各类灾害和突发事件应急预案，普及防灾减灾知识，提高自救互救能力。

　　加强城市精细化管理。搭建全方位数字城管平台，推进老旧管网升级改造，排除路面塌陷、管线破裂、燃气泄漏等安全隐患。进一步扩大道路清洗覆盖面。大力发展公共交通，试行换乘优惠，提高服务水平。综合治理违法建筑、占道经营、非法运输。逐步改造危旧房和预制板房。保护风景名胜、风貌建筑、古树名木，挖掘地域文化特质，提升历史文化街区。

　　提高新型城镇化水平。坚持城乡统筹、“四化”同步，抓好全市城镇化规划布局，完善小城镇建设试点，加快实现农业转移人口市民化。加强农村基础设施建设，改善农村生态环境、村容村貌，保护青山绿水，打造田园风光。构建农民收入来源多元机制，促进农民持续稳定增收。

　　（五）着力深化两岸融合，进一步发挥前沿平台作用

　　把握机遇、发挥优势，先行先试、主动作为，深入做好与台湾人民交流合作的各项工作。

　　增进经贸合作实效。加强与台湾自由经济示范区、金门免税岛的对接合作。加快建设两岸新兴产业和现代服务业合作示范区、两岸区域性金融服务中心、两岸贸易中心、闽台（厦门）文化产业园，着力引进台湾百大企业、创新型中小企业，促进两岸产业融合发展。

　　密切亲情纽带联系。常态化运营海峡邮轮航线，建设对台海运快件物流中心，吸引更多两岸民众和货物经厦往返。继续办好海峡论坛等重大活动，争取国家部委发布更多促进两岸交流合作政策措施，提升对台文化、教育、卫生等领域的合作实效。推进与台湾市县、基层之间交流往来，深化以宗亲、民俗为主要内容的民间亲情交流。加大对台资企业和创业项目的扶持力度，让台胞在厦门创业顺心、工作称心、生活舒心。

　　（六）着力促进公平均衡，进一步繁荣各项社会事业

　　围绕保住基本、确保公平、促进均衡、激发活力，提升公共服务水平，推动社会事业全面发展。

　　办好人民满意的教育。新建公办幼儿园20所、改扩建中小学53所，各增加学位5000个、23000个。健全随迁子女就学服务保障机制，完善农村义务教育学生营养膳食补助制度。推进素质教育，促进义务教育均衡发展。标本兼治减轻学生课业负担，促进青少年身心健康、体魄健壮。改进美育教学，提高学生审美和人文素养。促进普通高中多样化有特色办学。大力发展职业教育，增强服务产业能力。支持厦大、集大、理工、华大等高校内涵发展，加强重点学科和专业建设。

　　提高医疗卫生水平。支持社会资本兴办非营利性医疗机构，鼓励各类医疗机构开展多形式合作。推进五缘医院、翔安医院、集美新城医院等项目建设。新增一批基层医疗卫生站点。力争70%村卫生所能够提供中医药服务。基本建成健康医疗云平台，减少重复检查，有效为患者减轻痛苦、节省费用。做好人口计生工作，提高出生人口素质。

　　加快建设文化强市。大力发展公益性文化事业，实施文化惠民工程，倡导全民阅读，推动更多公共文化设施免费开放，健全覆盖城乡的公共文化服务体系。深化闽南文化生态保护实验区建设，继续推进鼓浪屿和闽南红砖民居申遗。繁荣发展哲学社会科学。鼓励创作更多文化精品。办好国际马拉松、厦金海峡横渡等重大赛会，加快水上运动中心二期等体育设施建设，促进全民健身和竞技体育同步发展。

　　扎实推进文明创建。培育和践行社会主义核心价值观，以新一轮文明城市创建为抓手，积极搭建群众广泛参与的活动平台，发挥道德典型引领作用，大力营造勤劳节俭、遵法守礼、敬老尊贤的社会风尚，提升城乡文明整体水平。壮大志愿者队伍，树立志愿服务品牌。整治出版物市场和校园周边环境，努力营造未成年人健康成长的社会氛围。落实优抚政策，推进军民融合发展，争创全国双拥模范城“九连冠”。

　　（七）着力保障改善民生，进一步维护社会和谐稳定

　　按照守住底线、突出重点、完善制度的思路，充分发挥社会政策托底功能，在保障民生和提升服务中加强社会建设。

　　多渠道促进就业创业。新增就业18万人。完善促进高校毕业生、农村转移劳动力、城乡困难人员、退役军人就业的体制机制。打造创业信息共享平台，扩大小额担保贷款发放范围，健全公共创业服务体系。推进职业培训和技能人才队伍建设，完善校企人才对接和劳务协作机制。加强劳动仲裁和劳动保障执法监察，构建和谐劳动关系。

　　全面增强社会保障能力。进一步完善基本养老、医疗、工伤、生育、失业保险政策。适时提高最低工资标准，全面推进工资集体协商。更好服务来厦劳动者，切实维护其合法权益。完善低保标准与物价上涨挂钩联动机制。发展养老服务业，支持新建扩建养老机构。加快保障性住房建设，健全准入和退出机制。加强粮食安全保障体系建设。推进“菜篮子”工程，新建一批直控基地。落实最严谨的标准、最严格的监管、最严厉的处罚、最严肃的问责，保障农产品质量和食品药品安全。

　　维护社会安定稳定。健全社会治安防控体系，严厉打击各种刑事犯罪，建设平安厦门。做好废止劳教制度后续相关工作，健全社区矫正制度。严格落实安全生产责任制，深化道路交通安全专项整治，加强网络安全防控。完善社会稳定风险评估和突发公共事件应急处置机制，提高公共危机管理和风险管控能力。加强信访工作，建立畅通有序的诉求表达、矛盾调处、权益保障机制。统筹推进、突出重点，创建全国和谐社区建设示范城市。

　　（八）着力生态文明建设，进一步推动城市可持续发展

　　生态环境既是生产力，也是竞争力。我们要把生态文明建设摆在更加突出的位置，自觉推动绿色发展、循环发展、低碳发展。

　　全面促进资源节约。加快经济社会发展规划、土地利用总体规划、城市总体规划“三规合一”，促进发展目标、用地指标、空间坐标“三标衔接”，优化国土空间开发格局，注重节约集约用地。推动重点领域节能降耗，实施节能产品惠民工程，发展绿色建筑。推广清洁能源和清洁生产，促进循环经济发展。

　　切实保护和改善环境。实施生态保护与重大生态修复工程，扩大森林、湖泊、湿地面积，保护生物多样性。加强饮用水源地保护，推进流域综合整治，强化陆源入海污染物防控，加快岛外污水处理设施和管网建设。开展清洁空气行动，淘汰尾气超标车辆。做好工地防尘降噪，制止土方车、垃圾车跑冒滴漏。实施立体绿化，做好村头村尾、房前屋后、道路两侧、溪流两岸的绿化工作，加快建设一批城乡公园，积极创建国家生态市和生态园林城市。

　　完善生态文明制度。严格实行主要污染物总量减排目标责任制，严肃生态环保责任追究，实施生态文明考核办法。降低产业能耗，严守环保准入门槛，推行低碳产品认证制度，建立产业升级和环境升级并重的发展模式。

　　三、不断加强政府自身改革和建设

　　全面深化改革、建设美丽厦门，要求我们进一步解放思想、与时俱进，锐意进取、开拓创新，全面承担起时代、人民赋予的崇高使命和重大责任。

　　大力推进投资环境国际化。这是经济特区必须率先作为的使命所在，是建设现代化国际性港口风景旅游城市的必然要求。我们要以改革创新精神，加快完善现代市场体系，更好地发挥市场在资源配置中的决定性作用；以扩大开放措施，倒逼形成与国际接轨的体制机制；以优质高效服务，强化市场监管、规范市场秩序、消除市场壁垒、保护知识产权；以公正、开明、包容、诚信的价值取向，营造城市功能完善、法规制度健全、公共服务优良的投资发展环境。

　　大力推进治理体系和治理能力现代化。改变理念，创新方法，重点加快建立政府主导、社会协调、公众参与的社会治理模式，实现政府治理与社会自我调节、居民自治良性互动。强化街道社会服务和社区自我管理、自我服务职能，构建多元主体参与的基层社会治理体系。更加重视制度治理作用，把制度优势转化为工作实效，实现各项事务治理的规范化、程序化。

　　进一步加强民主法治建设。主动接受市人大及其常委会的法律监督、工作监督，重大决策出台前向人大报告，重要事项提请人大讨论决定。自觉接受市政协的民主监督，拓展协商民主形式，认真听取政协委员、各民主党派、工商联、各人民团体和各界人士的意见建议。畅通民主渠道，从各层次各领域扩大公民有序政治参与。加强政府立法前期工作，完善规范性文件、重大决策合法性审查机制，加强对行政执法的监督，不断提高科学决策、民主决策和依法决策水平。

　　切实提高政府执行力。坚持求真务实，坚决纠正“四风”，改进文风会风，少开会、开短会，开解决实际问题的会；少讲话、讲短话，讲群众听得懂、记得住、用得上的话；少发文、发短文、发简明扼要管用的文。坚持“四下基层”，持续开展党的群众路线教育实践活动，到全面深化改革的主战场、建设美丽厦门的第一线、服务人民群众的最前沿，去推动工作、解决问题。坚持主动作为，在矛盾面前敢于碰硬，在困难面前敢于进取，在挑战面前敢于担当。坚持狠抓落实，强力推进“马上就办、办就办好”，以治庸强素质、以治懒增效率、以治散正风气，踏石留印、抓铁有痕，确保决策部署一经做出，就一抓到底、抓出成效。

　　持之以恒建设廉洁政府。树立正确的权力观、地位观、利益观，坚持自重、自省、自警、自励，克己奉公、勤勉尽责，做人民满意公仆。坚决贯彻中央八项规定，严明政治纪律，严守党纪国法，坚决落实厉行节约反对浪费等系列要求。坚持用制度管权管事管人，健全财务预算、核准和审计制度，严控“三公”经费支出和楼堂馆所建设。推进惩治和预防腐败体系建设，加大对重点领域的监管和风险防控，把权力关进制度的笼子里。加强行政监察，严肃查处各类违纪违法案件，切实做到干部清正、政府清廉、政治清明。

　　各位代表！梦想孕育奇迹，奋斗铸就辉煌。让我们紧密团结在以习近平同志为总书记的党中央周围，在市委的坚强领导下，凝心聚力，真抓实干，为谱写好中国梦的厦门篇章，为开创厦门经济特区更加灿烂美好的明天而努力奋斗！
